# Advances in the interrelated nature of vaginal microecology, HPV infection, and cervical lesions

**DOI:** 10.3389/fcimb.2025.1608195

**Published:** 2025-06-19

**Authors:** Mingyu Cui, Yishi Wu, Zongyu Liu, Yunfei Liu, Limei Fan

**Affiliations:** Department of Obstetrics and Gynecology, The Second Norman Bethune Hospital of Jilin University, Changchun, Jilin, China

**Keywords:** vagina, microecology, HPV, cervical lesions, cytokines

## Abstract

Vaginal microecology serves as a crucial defense mechanism in women’s reproductive health. It encompasses vaginal anatomy, microbial flora, endocrine regulation, and immune responses. *Lactobacillus* species dominate this ecosystem, maintaining a dynamic balance essential for vaginal health. Studies have highlighted a strong association between vaginal microecology, human papillomavirus (HPV) infection, and cervical lesions. A well-balanced vaginal microenvironment enhances mucosal barriers and immune function, aiding in HPV prevention and clearance. Conversely, disruptions in vaginal microecology compromise these defenses, increasing susceptibility to HPV infection. Persistent high-risk HPV (HR-HPV) infections are key contributors to cervical lesions and may further destabilize the vaginal microbiota(VMB). Additionally, cervical lesion progression is influenced by local immune responses, with HPV infection potentially accelerating disease development by suppressing cervical immunity. This review explores the intricate association between vaginal microecology, HPV infection, and cervical lesions, offering insights into early diagnosis, prevention, and treatment strategies.

## 1 Introduction

Cervical cancer (CC) is a common gynecological malignancy, ranking fourth among female cancer-related deaths worldwide (Pimple and Mishra, 2022). Latest data from the World Health Organization show that in 2022, approximately 660,000 new CC cases and 350,000 related deaths occurred globally (Organization W. H. Cervical cancer). China remains one of the countries with the highest CC incidence and mortality (Singh et al., 2023), with data from 2020 indicating approximately 110,000 new cases annually, accounting for 18.2% of global cases. In addition, approximately 59,000 women die from this disease every year, accounting for 17.3% of total global deaths (Zou et al., 2020), posing a substantial threat to women’s health. Unlike other malignancies, CC has a well-established etiology (Bornstein et al., 1995). Persistent infection with human papillomavirus (HPV), particularly high-risk HPV (HR-HPV), has been identified as the primary causative agent of CC and squamous intraepithelial lesions (SIL) (Rahangdale et al., 2022).

The mucosal immune system of the female genital tract, vaginal microbiota(VMB), and other host factors influence the persistence or clearance of HPV, thus concerning the risk of CC (Schellekens et al., 2025). In a healthy vaginal environment, microbial diversity is relatively low, with *Lactobacillus* being the predominant bacteria (Leon-Gomez and Romero, 2024). Lactobacilli maintain the vaginal microecological balance and produce lactic acid, hydrogen peroxide (H2O2), and bacteriocins, which effectively inhibit the overgrowth of pathogenic bacteria and strengthen the vaginal mucosal barrier. This protective mechanism reduces the likelihood of viral and bacterial infections and enhances local antimicrobial and anti-tumor defense capabilities (Xu et al., 2025). HPV infection disrupts the original acidic environment of the vagina, potentially triggering mucosal immune responses and genital inflammation, which, in turn, alters the VMB (Gardella et al., 2022). Vaginal microecological imbalance facilitates HPV adhesion, impairs cervical immune defenses, and promotes the invasion and colonization of pathogenic bacteria. This vicious cycle elevates vaginal pH and shifts the microbial community away from *Lactobacillus* dominance, leading to chronic inflammation, persistent HPV infection, and disease progression, finally increasing CC risk (Lin et al., 2022).

Despite the growing evidence of the association among vaginal microbiota, host immune response, and HPV infection, the underlying mechanism of their interaction remains elusive. Further investigation into the roles of immune regulation and vaginal microecology in HPV infection is essential for elucidating viral persistence and developing more effective prevention and therapeutic strategies. This review looks into the role of vaginal microecology in the development of HPV-related cervical diseases. It provides theoretical support for early diagnosis, future interventions, and microbiota-targeted prevention measures.

## 2 Vaginal microecology

### 2.1 Definition and composition

The female vagina is a dynamic yet relatively stable ecosystem encompassing the VMB, host endocrine system, vaginal anatomy, and local immune defenses (Qing et al., 2024). The vaginal microbiota constitutes the core of this microecosystem. As an open cavity, the vagina is colonized by various microorganisms, such as bacteria, fungi, and viruses (Ye and Qi, 2024). These microorganisms primarily reside in the vaginal mucosal epithelium and form biofilms through hierarchical and structured colonization. The microbial composition within these biofilms undergoes constant succession in response to physiological and environmental changes (Usyk et al., 2020). The vaginal flora forms a symbiotic relationship with the host, supporting normal physiological functions and ensuring reproductive health.

### 2.2 Functions of vaginal microecology

In women of reproductive age, the VMB is both vast and complex. It is estimated that the total bacterial load usually ranges from 10¹^0^ to 10¹¹. Among these, *Lactobacillus* is the predominant genus, accounting for over 70% of the total bacteria. *Lactobacillus* plays a crucial role in maintaining vaginal microbiota balance, curbing the reproduction of pathogenic microorganisms, strengthening local immune defenses, and providing anti-tumor protection (Shen et al., 2024). The key mechanisms by which these beneficial bacteria contribute to health include:

① Lactobacilli produce lactic acid to maintain vaginal acidity. They derive energy from the carbohydrates released by vaginal mucosal epithelial cells. Lactobacilli metabolize glycogen into lactic acid, which creates an acidic environment (pH < 4.5) that significantly inhibits the adhesion, colonization, and proliferation of pathogenic bacteria (Shen et al., 2024).② Lactobacilli secrete H_2_O_2_, bacteriocins, and other compounds with antibacterial efficacy. H_2_O_2_ increases cell membrane permeability by generating highly reactive hydroxyl radicals, thereby preventing the invasion of pathogens into cervical epithelial cells (Frąszczak et al., 2022). Additionally, Lactobacilli secrete bacteriocins and biological surfactants, which are antimicrobial peptides or proteins that disrupt epithelial cells and form a frontline defense against pathogen adhesion (Borgogna et al., 2020; Nieves-Ramírez et al., 2021). ③ Lactobacilli prevents pathogenic microorganisms from adhering to vaginal epithelium by competitively binding to mucosal epithelial receptors. Furthermore, it secretes peptidoglycans and extracellular polypeptidoglycans (EPS) to form a biofilm with physical barrier functions to reduce pathogen colonization (Kalia et al., 2020). Different *Lactobacillus* species secrete distinct protective substances (Wang et al., 2019a). For example, *Lactobacillus crispatus* (*L. crispatus*), a predominant vaginal species, produces adhesion factors that facilitate mucosal colonization and inhibit *Gardnerella vaginalis* adhesion. In contrast, *Lactobacillus iners*(*L. iners*) lacks this protective effect (Łaniewski et al., 2019). Women with *L. iners*-dominant microbiota experience a three to five times higher risk of HPV infection and a two to three times greater likelihood of HR-HPV progression, cervical dysplasia, or cancer than women with *L. crispatus*-dominant microbiota (Palma et al., 2018). This highlights the superior role of *L. crispatus* in vaginal defense. ④ Lactobacilli enhance immune function by activating T-cell proliferation and differentiation, ameliorating the immunological recognition and proliferation of B cells (Frąszczak et al., 2022). Lactic acid suppresses toll-like receptor agonists, lowering pro-inflammatory cytokines, such as interleukin-6 (IL-6), tumor necrosis factor (TNF), RANTES, IL-8, and macrophage inflammatory protein 3α (MIP3α). This anti-inflammatory effect helps shield genital tract epithelial cells from infections and damage (Hearps et al., 2017).

### 2.3 Classification of vaginal microbiota

Currently, most domestic and international scholars agree that the VMB can be classified into five community state types (CSTs), with dominant bacterial groups identified for each type (McClymont et al., 2022; Peremykina et al., 2024). *L. crispatus*, *Lactobacillus gasseri*(*L. gasseri*), *L. iners*, and *Lactobacillus jensenii*(*L. jensenii*) prevail in CSTs I, II, III, and V, respectively, whereas CST IV is characterized by increased microbial diversity, marked by reduced *Lactobacillus* abundance and a higher prevalence of anaerobic bacteria. CST IV is further subdivided into: CST IV-A (comprising the *Anaerococcus*, *Peptoniphilus*, *Corynebacterium*, *Prevotella*, *Finegoldia*, *Streptococcus*); CST IV-B (including the *Atopobium*, *Fannyhessea*, *Gardnerella*, *Sneathia*, *Mobiluncus*, *Megasphaera*); and CST IV-C, which features other diverse anaerobic species (France et al., 2020). Notably, differences in vaginal microenvironments across CSTs may directly affect HPV susceptibility and persistence.

## 3 Abnormal vaginal flora composition is associated with cervical HPV infection and cervical lesions

A balanced VMB plays a critical role in preventing infections of the female reproductive tract. Disruptions in microbiota composition are closely related to the development of cervical lesions (Žukienė et al., 2025). Collectively, vaginal dysbiosis may act as a cofactor for HPV infection. Investigating the interaction between VMB and HPV may enhance the understanding of HPV pathogenesis and facilitate the development of novel approaches for preventing cervical lesions. Common infections of the female urogenital tract include bacterial vaginosis (BV), vulvovaginal candidiasis (VVC), aerobic vaginitis (AV), and sexually transmitted infections(STIs).

**Table 1** summarizes the clinical research on VMB, HPV infection, and cervical lesions.

**Table 1:** Relationships between abnormal vaginal flora findings, HPV infection, and cervical lesions.

Abnormal vaginal flora	Detection method	Risk of HPV infection (increased/ irrelevant/ decreased)	Association with HPV infection	Risk of cervical lesions (increased/irrelevant/reduced)	Relationship with cervical lesions	Research type	Relevant references
BV	Metagenomics	Increase	Women with BV (who usually have a lower abundance of lactobacilli) are more likely to be infected with HPV	Increase	BV increases the risk of cervical lesions by upsetting the balance of the vaginal microbiome, increasing the growth of harmful flora and triggering an inflammatory response	Predictive function analysis	(Onywera et al., 2021)
	16S rRNA Sequencing	Increase	In the HPV-infected state, BV may exacerbate vaginal microecological dysregulation as evidenced by increased microbial diversity and enrichment of specific species (e.g., L. iners)	Increase	BV is a risk factor for CIN and may contribute to the development of cervical lesions by affecting the vaginal microecological balance	Cross-sectional analysis	(Xu et al., 2022)
VVC	Vaginal microbiological test	Decrease	VVC can reduce the risk of infection with other subtypes of HPV	Decrease	Candida albicans is a potential immunotherapeutic agent that could be used to develop new vaccines or treat HPV infections or other diseases	Cross-sectional analysis	(Feng et al., 2023)
	Vaginal microbiological test	Irrelevant	VVC is not associated with HPV infection				(Meng et al., 2016)
	PCR	Increase	VVC is a high-risk factor for HPV infection. When lactobacilli are dysregulated, the incidence of VVC and recurrent VVC infections increases dramatically	Increase	The detection rates of Candida in chronic cervicitis, CIN I, CIN II-III, and cervical cancer were 17.54%, 25.00%, 39.13%, and 62.50%, respectively, suggesting that Candida may increase the risk of cervical lesions	Case analysis studies/retrospective studies	(Tian et al., 2022b)
AV	Wet sheet microscopy	Increase	There was a positive correlation between the severity of AV and the severity of cervical HPV-induced lesions	Increase	Moderate to severe AV is strongly associated with CIN2+	Case-control study	(Plisko et al., 2021)
	Cervical smear + vaginal PH + microscope	Irrelevant	AV is not an indicator of HPV infection	Increase	AV is very common in patients with low-grade squamous intraepithelial lesions	Prospective study	(Jahic et al., 2013)
TV	Vaginal microecological test +TCT	Increase	TV is associated with HR-HPV infection (P < 0.0001)	Increase	TV mixed infections increased the risk of CIN 1 in female patients with HR-HPV and increased the risk of CIN 2-3 in female patients with HPV 16	Cohort study	(Yang et al., 2020)
		Irrelevant	No association shown between TV and HPV infection	Irrelevant	No significant correlation between TV and CIN	Meta-analysis	(Liang et al., 2019)
	Swab culture			Irrelevant	The effect of TV infection alone on the risk of persistence or progression of CIN1/L-SIL was not significant.	Retrospective cohort study	(Raffone et al., 2020)
CT	PCR	Increase	Increased (when co-infected with Chlamydia trachomatis)	Increase	Co-infection of Chlamydia trachomatis with HPV may indirectly affect the risk of cervical lesions by increasing the risk of HPV infection		(Chen et al., 2020)
	Vaginal microbiological test	Irrelevant	Chlamydia trachomatis infection did not increase the risk of HPV infection or cervical lesions	Irrelevant	There was no significant association between Chlamydia trachomatis infection and the severity of cervical lesions	Cross-sectional study	(Jiang et al., 2023)
UU	PCR			Increase	Mh and Uu co-infection is associated with increased risk of cervical intraepithelial neoplasia (CIN) grade 3 and invasive cervical cancer	Cross-sectional study	(Adamopoulou et al., 2021)
	PCR	Partial increase	U. parvum serotypes 1, 3, and 6 were associated with HPV infection. However, after adjusting for other STIs, no significant correlation was observed between U. parvum serotype 14 and HPV infection	Increase	Certain Ureaplasma urealyticum serotypes (notably U. parvum serotype 6) have been identified as independent risk factors for the development of CIN	Multi-center, cross-sectional study	(Zhang et al., 2025)
*N. gonorrhoeae*	Nucleic acid extraction+*NG* assays	Increase	Co-infection of *NG* and HPV exists among gynecological outpatients. *NG* infection also increases the risk of HPV infection	Increase	While NG-HPV co-infection's statistical significance may be sample-limited, the observed association suggests mixed infections may elevate cervical lesion risk	Cross-sectional study	(Lu et al., 2023)
	Detection of sexually transmitted pathogens	Increase	Among patients infected with HR-HPV (such as HPV 16/18), the co-infection rate of *NG* was significantly higher than in those infected with other HPV types			Cross-sectional study	(A et al., 2023)
HSV	qPCR+MSD+16S rRNA	Increase	Co-infection with HSV-2 and HPV exists among young women, and the state of the vaginal microbial community may influence the interaction between HSV-2 and HPV.	Increase	Reactivation of HSV-2 could affect HPV persistence or viral load, potentially influencing the progression of cervical lesions.	Case Report+Longitudinal Study	(Uysal et al., 2022)
HIV	Multi-omic techniques+experimental models	Increase	HIV-positive individuals exhibit a higher prevalence of HPV infection. HIV compromises mucosal immunity, increasing the risk of persistent HPV infection.	Increase	Among people living with HIV, CIN may progress more rapidly and is more likely to develop into CC. The immunodeficiency caused by HIV may facilitate more efficient integration of HPV into the host genome, accelerating carcinogenesis.	Review+basic research	(Moreno et al., 2023)

BV, Bacterial Vaginosis; VVC, Vulvovaginal candidiasis; AV, Aerobic vaginitis; TV, Trichomonas vaginitis; PCR, Polymerase Chain Reaction; TCT, Polymerase Chain Reaction; CIN, Cervical Intraepithelial Neoplasia; L-SIL/LG SIL, low-grade squamous intraepithelial lesion; *N. Gonorrhoeae*/NG, *Neisseria gonorrhoeae;* HSV, herpes simplex virus; HIV, Human Immunodeficiency Virus; CC, cervical cancer.

### 3.1 Association of BV with HPV infection and cervical lesions

Numerous studies have demonstrated a strong correlation between BV and HPV infection, with BV recognized as an independent risk factor for HPV acquisition and cervical lesions (Xu et al., 2022; Paul et al., 2023). CST III-B, IV-A, and IV-B are prevalent in patients with BV (Dong et al., 2024). BV is commonly caused by pathogens, including *Gardnerella*, *Prevotella*, *Campylobacter*, *Bacteroides*, *Atopobium vaginae*, and *Sneathia*. A meta-analysis encompassing six studies further confirmed the positive association between BV and cervical HPV infections (Martins et al., 2023). Similarly, another study used 16S rRNA gene sequencing to analyze the association between BV, HPV infections, and cervical lesions (Wei et al., 2020). HR-HPV-positive individuals exhibited decreased levels of *Lactobacillus* and elevated proportions of BV-associated bacteria, such as *Gardnerella*, *Prevotella*, *Fusobacterium*, *Actinomyces*, *Peptococcus*, *Anaerococcus*, *Peptostreptococcus*, *Streptococcus*, and *Ureaplasma urealyticum*. These results underscore a strong association between BV and HR-HPV infection. Dong et al. conducted a 2-year longitudinal study involving reproductive-aged women, demonstrating that BV-positive individuals showed significantly higher rates of persistent HR-HPV infection than BV-negative individuals (Dong et al., 2022). Through combined 16S rRNA sequencing and quantitative reverse transcription polymerase chain reaction analysis of vaginal secretions and cervical cells, vaginal *Prevotella* overgrowth was found to activate the NF-κB/C-Myc signaling pathway, facilitating HR-HPV persistence and cervical lesion progression. This effect may be further amplified by sialidase secretion. Microbial infection-induced NF-κB activation stimulates C-Myc expression, which in turn upregulates hTERT to drive malignant transformation (Papanikolaou et al., 2011; Ghareghomi et al., 2021). Lam et al. proposed that intratumoral microbiota may contribute to cervical carcinogenesis through immune modulation. They specifically suggested that *Prevotella bivia(P. bivia)* upregulate the human cancer driver lysosome-associated membrane protein 3 (LAMP3), which promotes metastasis and may help eliminate episomal HPV. This process can lead to overexpression of the E6 and E7 HPV oncogenes, thereby accelerating cervical disease progression (Lam et al., 2018).

BV may provide a biological rationale for HPV infection and invasion. However, Mao et al. identified a temporal sequence between HPV and BV infections, with HPV infection generally preceding BV. This may be attributed to the imbalance in the vaginal microenvironment caused by HPV infection, which increases the likelihood of BV (Mao et al., 2003). Therefore, the direct association between BV and cervical HPV infection, whether BV infection disrupts vaginal microecology and increases the prevalence of HPV infection and cervical lesions, whether HPV infection induces changes in the vaginal microecology that lead to BV infection, or whether these conditions are interdependent and promote simultaneous infections remains unclear. A substantial number of epidemiological and molecular studies are required to further explore the association between HPV infection and cervical lesions. Additionally, further research on the interaction between HPV infection and BV may facilitate the use of simple vaginal microecology tests, such as pH measurement, Gram staining for Nugent scoring, or molecular assays (e.g., quantitative PCR or 16S rRNA sequencing) targeting key bacteria (e.g., *Lactobacillus* spp., *Gardnerella vaginalis*, and *Atopobium vaginae*). These tests may help assess vaginal dysbiosis and predict HPV susceptibility. For instance, a low *Lactobacillus* dominance combined with a high anaerobic bacterial load may serve as a practical biomarker for increased HPV risk. Such approaches, if validated, might be integrated into routine gynecological screening to improve early detection and prevention strategies.

### 3.2 Association of VVC with HPV infection and cervical lesions

VVC is a common infectious disease of the lower genital tract caused by *Candida albicans*, which is a conditionally pathogenic fungus that causes disease only when the local immune capacity of the body or vagina declines (Sobel and Vempati, 2024).

The correlation between VVC, HPV infection, and cervical lesions remains controversial. Some researchers pose that VVC increases susceptibility to HPV and hinders HPV clearance (Wang et al., 2020b; Wu and Xue, 2020). This may result from pathogen-secreted proteolytic enzymes that activate the complement cascade, generating anaphylatoxins and chemokines. These factors cause local vasodilation, increased permeability, and an inflammatory response, finally inhibiting chemotaxis and the activation of neutrophils and lymphocytes (Ghosh et al., 2016). Additionally, VVC produces invasive enzymes that can damage genital epithelial cells, potentially facilitating HPV adhesion and persistence by creating a favorable microenvironment for viral replication (Wang et al., 2024). However, VVC does not raise the risk of HPV infection, and having both VVC and HPV does not lead to more severe cytological abnormalities (Wang et al., 2020b; Long et al., 2023). Furthermore, most women with VVC have a vaginal pH below 4.5; this acidic environment enhances vaginal defense by suppressing pathogen survival (Kwon and Lee, 2022). The low pH further bolsters immune responses by promoting the production of antimicrobial peptides (e.g., defensins) and lactic acid, which inhibit viral replication and maintain epithelial barrier integrity (Czechowicz et al., 2022). Consequently, VVC may confer a protective effect against persistent HPV infection, potentially reducing the risk of cervical intraepithelial lesions. Smalley et al. found that VVC may lower the risk of infection from non-16/18 HPV subtypes. Moreover, VVC functions as a possible booster for HPV vaccines because it may stimulate T-cell activity and improve immune function (Smalley Rumfield et al., 2020). This presents new avenues for vaccine and immunotherapy development. While numerous clinical studies have investigated the association between VVC and HPV infection/cervical lesions, substantial heterogeneity exists across study populations, including both general and high-risk groups. For instance, some studies enrolled balanced cohorts of premenopausal and postmenopausal women, whereas others specifically focused on HPV-vaccinated individuals. These demographic variations (e.g., age, immune status, and geographic distribution) may account for the inconsistent conclusions regarding the VVC-HPV association. Future investigations should utilize stratified analyses controlling for these covariates to elucidate potential confounding effects.

### 3.3 Association of AV with HPV infection and cervical lesions

In 2002, Donders et al. introduced the concept of AV based on its bacteriological, immunological, and clinical characteristics (Donders et al., 2002). Similar to BV, AV is characterized by a reduction in H_2_O_2_-producing *Lactobacillus* species or a decrease in *Lactobacillus* activity within the vaginal microenvironment. However, unlike BV, AV is associated with an overgrowth of aerobic bacteria, primarily *Streptococcus*, *Staphylococcus*, and *Escherichia coli*, which are compositionally aligned with CST IV (Zeng et al., 2023). Because of the relatively recent clinical recognition of AV, studies investigating its association with HPV infection and CC remain limited. Jahic et al. conducted a prospective study and reported that AV was significantly more prevalent in women with cervical intraepithelial lesions than in those with healthy cervical cytology (Jahic et al., 2013). Furthermore, AV treatment appeared to promote the regression of cervical precancerous lesions. The proposed mechanism suggests that AV disrupts vaginal microecology by reducing *Lactobacillus* populations, thereby increasing the vaginal pH. The loss of *Lactobacillus*, the dominant protective bacterium, weakens the defense against external pathogens, leading to leukocytosis and enhanced interstitial invasion of cervical tissue by inflammatory cells, particularly through leukocyte esterase activity (Donders, 2007; Wang et al., 2020a). Vieira-Baptista et al. reported that moderate-to-severe AV was independently associated with an increased risk of cervical cellular abnormalities, despite no direct correlation with cervical HPV infection (Vieira-Baptista et al., 2016). Considering the limited national and international research on AV and HPV, further large-scale studies are required to elucidate their association.

### 3.4 STIs

#### 3.4.1 TV

TV is a lower genital tract infection caused by *Trichomonas vaginalis*, a prevalent sexually transmitted pathogen. The parasite secretes proteases, consumes or phagocytoses glycogen from vaginal epithelial cells, and inhibits lactic acid production, increasing the vaginal pH. Additionally, it consumes oxygen, creating an anaerobic environment that favors the proliferation of anaerobic bacteria (Li et al., 2022).

There are inconsistent findings about the association between TV and HPV infection and cervical lesions. Belfort et al. reported that TV is associated with an increased risk of HR-HPV infection, with TV-positive patients exhibiting a higher risk of HR-HPV infection than TV-negative patients (Belfort et al., 2021). This may be attributed to the depletion of *Lactobacillus* populations and subsequent reduction in lactic acid secretion in patients with TV, leading to vaginal microecological imbalances, increased inflammatory factor secretion, and reduced local cervical immunity (Mei et al., 2023). Yang et al. concluded that TV is significantly associated with HPV infection, proposing that flagellated protozoa attach to epithelial cells and induce toxic reactions, thereby increasing HPV infection risk. Moreover, TV induces a sustained inflammatory response in the cervix and vagina, damaging the cervical epithelium and accelerating the erosive effects of HPV on the cervix (Yang et al., 2020). However, Li et al. suggested that HPV infection may prevent TV infection (Li et al., 2022). HPV infection activates the immune response, triggering the release of immune cells and factors that provide localized immunity against TV. Additionally, Feng et al. examined 25,054 women and reported that although TV-positive women had a higher risk of HR-HPV infection, they exhibited a decreased risk of developing cervical intraepithelial neoplasia grade 2 or higher (CIN2+) (Feng et al., 2018). However, other studies report no strong association between TV and HPV. For example, Liang et al. found no association between these two infections (Liang et al., 2019). Similarly, Raffone et al. observed that TV infection alone did not significantly affect HPV rates (Raffone et al., 2020). These inconsistent findings may stem from differences in study populations and sample sizes. This necessitates large-scale clinical studies to clarify the association between trichomoniasis, HPV infection, and cervical lesions.

#### 3.4.2 CT and UU

*Chlamydia trachomatis* (CT) and *Ureaplasma urealyticum* (UU) infections represent clinically prevalent urogenital diseases transmitted primarily through sexual contact (Liu et al., 2024). The association of CT and UU infections with the progression of HPV infection and cervical lesions remains debatable. A meta-analysis by Liang et al. suggested that CT infection raises the likelihood of HPV infection. One possible explanation is that CT attaches to the genital mucosa, disrupts lysosomal activity in host cells, and causes microdamage and localized inflammation. This compromises immune defenses of the cervix and vagina, thus increasing susceptibility to HPV and potentially accelerating CIN and CC development (Liang et al., 2019). In contrast, Wang et al. reported no significant correlation between CT and HR-HPV or cervical lesions, despite a moderately higher prevalence of CT infection in HPV-positive cases (Wang et al., 2019b). Similarly, Abreu et al. suggested that CT positivity does not increase the risk of CC but may be associated with LSIL and HSIL (de Abreu et al., 2012). Conversely, other studies found no significant association between HPV infection and CT (Meng et al., 2016).

Researchers have demonstrated significantly higher UU prevalence in HPV-positive groups, establishing a significant association between UU and HPV infections (Lu et al., 2023). UU may trigger viral persistence and cellular abnormalities, acting as a cofactor in HPV-induced precancerous cervical lesions and CC (Plummer et al., 2021). One possible explanation is that mycoplasma infection induces the release of pro-inflammatory cytokines from cervical macrophages, disrupting the mucosal barrier of the cervix. This results in localized congestion, epithelial cell degeneration, necrosis, and periungual inflammatory infiltration of the mucosa, submucosal tissues, and glands (Lv et al., 2019; Liu et al., 2021). Additionally, UU can adhere to host cells and produce phospholipases that degrade host cell membranes, altering cellular functions. UU breaks down urea, releasing toxic ammonia that damages cells, whereas its immunoglobulin A (IgA) proteases degrade mucosal IgA, impairing immune defenses and facilitating HPV invasion and colonization (Chen et al., 2014; Adebamowo et al., 2017). However, other studies have reported no significant correlation between UU infection and HR-HPV infection (Zhang et al., 2017). Hence, larger sample sizes and long-term follow-up studies are necessary to clarify the association and underlying mechanisms.

#### 3.4.3 *N. gonorrhoeae* and HSV

*Neisseria gonorrhoeae* (*N. gonorrhoeae*) and herpes simplex virus (HSV) are common sexually transmitted pathogens. Epidemiological studies indicate a high co-infection rate of *N. gonorrhoeae*, HSV, and HPV among sexually active populations, likely associated with high-risk sexual behaviors (e.g., unprotected intercourse, multiple partners). Moreover, these pathogens may act synergistically to significantly increase the risk of malignancies, such as cervical and anal cancers (Klein et al., 2024). Co-infections involving HR-HPV and non-HPV STIs (e.g., *N. gonorrhoeae*, HSV-2) have been related to HPV persistence, cervical dysplasia, and neoplastic progression (Ma et al., 2022). *N. gonorrhoeae*-HPV co-infection may elevate CC risk, necessitating enhanced clinical surveillance and prevention of STIs like *N. gonorrhoeae* (Latorre-Millán et al., 2025). Notably, HSV-2 is significantly more prevalent among HPV/HR-HPV-positive women (Klein et al., 2024). However, other research has reported a higher HSV-1 seropositivity rate in HPV-positive women than in HPV-negative individuals, suggesting a possible synergistic role of HSV-1 with HPV in increasing the risk of CIN, whereas the impact of HSV-2 remains unclear (Finan et al., 2006). The underlying mechanisms may involve genital mucosal inflammation and local immune suppression induced by *N. gonorrhoeae* and HSV, facilitating poly-microbial co-infections and prolonged pathogen persistence (Quillin and Seifert, 2018). Furthermore, treatments for *N. gonorrhoeae* or HSV (e.g., antibiotics/antivirals) may alter vaginal/cervical microbiota, thus indirectly influencing HPV infection outcomes (Sausen et al., 2023).

#### 3.4.4 HIV

Human Immunodeficiency Virus (HIV) and HPV are both sexually transmitted pathogens and share a complex epidemiological association and biological interaction. A meta-analysis of HPV infection among HIV-infected individuals in China reported an HPV infection rate of 52.54% (Yuan et al., 2023). A systematic review indicated that the infection rate of high-risk HPV (HPV16, HPV18) in HIV-positive individuals was significantly higher than in HIV-negative individuals, and this co-infection status accelerated the progression of CIN to CC (Swase et al., 2025). Cambrea et al. examined HIV-positive women in southeastern Romania and suggested that HPV types 31 and 56 were more prevalent (Cambrea et al., 2022). Pavone et al. stated that HIV infection reduces helper T (CD4^+^ T) cells, weakening the immune response against HPV. The impaired function of dendritic cells (DCs) during co-infection further affects antigen presentation and T-cell activation, thus promoting persistent HPV infection. HIV-induced immunosuppression enhances the carcinogenic effects of HPV oncoproteins, such as E5, E6, and E7, which interfere with cell cycle regulation, promote cell proliferation, and inhibit apoptosis, thereby accelerating the malignant transformation of cervical epithelial cells. Additionally, HIV infection induces epithelial-mesenchymal transition (EMT) in cervical epithelial cells through the actions of gp120 and Tat proteins, promoting tumor cell invasion and metastasis. The EMT process involves the activation of multiple signaling pathways, such as mitogen-activated protein kinase and transforming growth factor-beta (TGF-β), which are closely related to the carcinogenic effects of both HIV and HPV (Pavone et al., 2024). Additionally, HPV infection may increase the risk of HIV acquisition through multiple mechanisms. First, HPV-induced inflammation leads to elevated levels of cytokines (such as IL-1, IL-6, IL-8, and TNF-α) and chemokines (such as MCP-1 and IP-10) in the genital tract. These mediators recruit more immune cells to the genital mucosa and may also disrupt the mucosal barrier, facilitating HIV entry. Because CD4^+^ T cells are the primary targets of HIV, their increased numbers directly elevate the risk of HIV infection. HPV infection may modulate immune responses by affecting the Toll-like receptor (TLR) signaling pathway. For example, the HPV E7 protein can recruit histone-modifying enzymes to suppress TLR9 transcription, weakening antiviral immune responses and facilitating HIV infection. Furthermore, HPV infection may alter the composition of the genital microbiota, characterized by a reduction in beneficial bacteria and an increase in harmful bacteria. This microbial imbalance may further exacerbate inflammation and increase the risk of HIV acquisition (Zayats et al., 2022; Swase et al., 2025).

Regarding HPV-HIV co-infection, researchers have proposed targeted prevention and treatment strategies (Arnold et al., 2022; Yuan et al., 2023). For example, strengthening HPV screening and preventive vaccination can reduce HPV infection rates, thereby lowering the risk of HIV acquisition and CC incidence. Meanwhile, for HIV-infected individuals, the early initiation of antiretroviral therapy helps restore immune function and reduces the risk and persistence of HPV infection.

## 4 Vaginal microecological functions and the role of HPV infection in cervix-associated diseases

**Figure 1:**
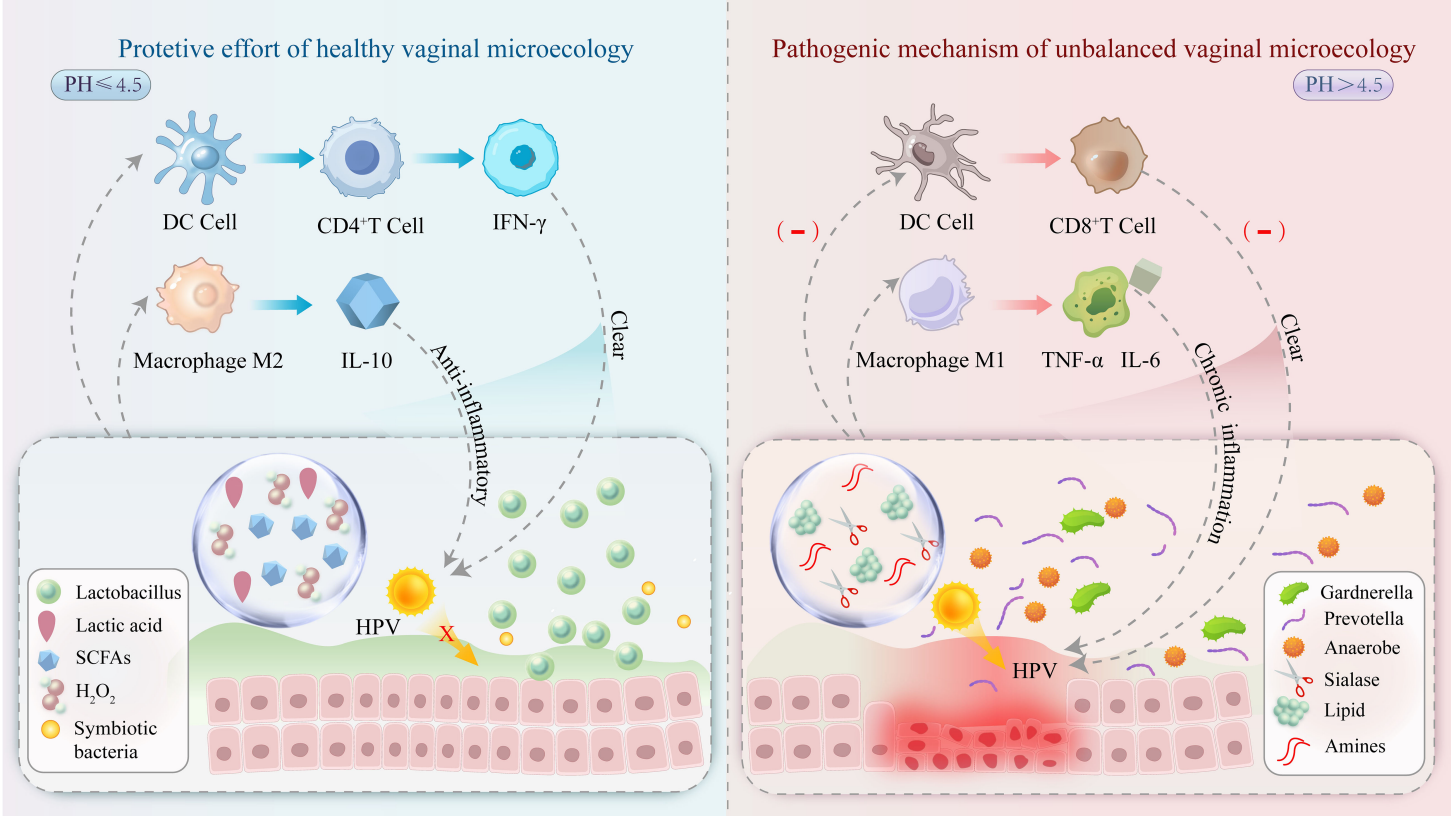
A healthy vaginal microecological niche (left) is dominated by *Lactobacillus* species, which maintain an acidic environment (pH ≤ 4.5) by secreting lactic acid, inhibiting HPV virus adsorption, and enhancing CD4^+^ T-cell activity to promote viral clearance. In the imbalanced microecological niche (right), pathogenic bacteria proliferate, and their metabolites (e.g., salivary acid lyase) disrupt the epithelial barrier and promote HPV invasion. In addition, DC cell function is inhibited, CD8^+^ T cell activity is reduced, and macrophage M1 polarization occurs with chronic inflammation leading to abnormal cell proliferation and promoting cervical lesion progression.

The interaction between HPV and vaginal microecology is a prominent research focus in gynecology. An imbalance in vaginal microecology-particularly a reduction in *Lactobacillus* populations-may elevate the risk of HPV infection. An altered VMB may contribute to the persistence of HPV infection and its progression to malignancy. Possible mechanisms include changes in the local immune response, microbial metabolite activity, and disruption of the epithelial barrier (**Figure 1**). Therefore, maintaining a balanced vaginal microenvironment may facilitate preventing HPV infection and its associated diseases.

### 4.1 Impact of cervicovaginal microecological dysregulation on mucosal barrier disruption

The mucosal barrier serves as the first line of immune defense against HPV infection, protecting against harmful environmental factors, including pathogens, while permitting symbiosis with mucosal microorganisms. The vaginal mucosal layer consists of a non-keratinized stratified squamous epithelium with numerous transverse folds. This physiological and anatomical structure functions as a natural defense barrier for the female reproductive system. HPV infection may colonize these vaginal wall folds, making viral clearance more challenging. Additionally, the epithelium contains innate immune cells that express Fc receptors, which bind to the Fc region of immunoglobulins, facilitating antibody-dependent protective functions. Particularly, macrophages and neutrophils express Fc receptor common γ-chain and Fc receptor common α-chain, respectively, allowing them to phagocytose pathogens coated with IgG and IgA (Anderson, 2022). However, when the vaginal mucosa is ruptured, particularly in the squamous-columnar junction zone (transformation zone) of the cervix, which is a preferred site for HPV because of its epithelial characteristics, the virus is more likely to invade to basal cells and integrate into their nuclei. This results in host cell genome alterations and the development of cervical lesions (Boda et al., 2018). Furthermore, dysbiosis of vaginal microorganisms may disrupt epithelial cell proteins and increase cell death, thereby facilitating HPV entry into cervical transformation zone epithelial cells, where the virus can replicate and progress to CIN (Barros et al., 2018). Lactobacilli help maintain an acidic environment and preserve the mucosal barrier by producing metabolites, such as lactic acid, bacteriocins, and biosurfactants, thereby protecting vaginal health and preventing pathogenic infections (Shen et al., 2024). Secretory leukocyte protease inhibitor (SLPI) is a low-molecular-weight protein with antimicrobial, anti-inflammatory, and anti-protease properties. It is secreted by keratinocytes-key targets of HPV infection-and contributes to cervical mucosal immunity (Zhang et al., 2023). Patients with BV exhibit decreased SLPI levels in vaginal secretions, thereby diminishing HPV inhibition (Miquel et al., 2022). Additionally, alterations in the vaginal microbial community reduce *L. crispatus* and D-lactic acid levels, allowing other bacteria to proliferate rapidly. This raises microbial diversity, expands anaerobic populations, and increases L-lactic acid. Consequently, the expression of extracellular matrix metalloproteinase inducer is enhanced, which activates extracellular matrix metalloproteinase-8 (MMP-8). MMP-8 breaks down the extracellular matrix and cytoskeletal proteins, weakening epithelial structure and accelerating cell death and desquamation. Upon HPV infection, the virus binds to heparan sulfate proteoglycans on basal keratinocytes via its L1 protein, entering through endocytosis before reaching the nucleus in vesicles (Rebolj et al., 2019). In patients with BV, elevated anaerobes and their metabolites, such as porotoxins and sialidase, heighten the activity of mucin-degrading enzymes. This enzymatic activity degrades the protective cervical mucus layer, thereby compromising vaginal epithelial integrity and enhancing viral adhesion, invasion, and genome integration. These effects finally increase cervical susceptibility to HPV (Muzny et al., 2020; Liu et al., 2023). Moreover, clinical proteomic and transcriptional studies have demonstrated that vaginal microbiome alterations lead to significant proteomic changes. These include cytoskeletal modifications (elevated actin histamine, reduced keratin, and keratinized envelope proteins), increased pro-inflammatory cytokine expression, enhanced proteolysis, decreased IgG1/2 levels, antimicrobial peptide imbalances, and altered mucous composition (Borgdorff et al., 2016). Upregulation of cytokine expression strongly correlates with reduced levels of neutrophil proteases (MMP-8 and MMP-9), decreased antiprotease levels, and disruptions in cytoskeletal organization, epithelial differentiation, and keratinization pathways (Mohammadi et al., 2022).

### 4.2 Localized cervicovaginal immunity and HPV infection

Most women are able to clear HPV infections through immune surveillance and defense mechanisms, thus preventing persistent infection. The immune system consists of two major components, namely innate immunity and adaptive immunity, both of which coordinate and function together to defend against and clear HPV (Gu et al., 2024).

#### 4.2.1 Innate immunity

Innate immune cells, including neutrophils, monocytes, macrophages, eosinophils, mast cells, and DCs, recognize and respond to invading pathogens through pattern-recognition receptors, such as TLRs, nucleotide oligomerization domain-like receptors (NLRs), and retinoic acid-inducible gene-like receptors (Lo Cigno et al., 2024). BV and its associated pathogens, such as Prevotella and Gardnerella, have been related to the expression of TLRs and NLRs, particularly TLR2 (Dong et al., 2022; Gerson et al., 2022). BV-related bacteria can induce immune responses in cervical cells through the TLR2-activated signaling pathway (Anton et al., 2022).

DCs are the most powerful antigen-presenting cells (APCs), and Langerhans cells (LCs) represent a key subset of DCs. LCs directly engage with HPV proteins in epithelial cells (Vine et al., 2024). HPV16 infection can reduce E-cadherin expression in infected keratinocytes, resulting in the depletion of LCs, thereby impairing the initiation of an effective immune response, which promotes persistent viral infection (Jackson et al., 2019). Additionally, macrophages play varied roles in immunity, influenced by their polarization into either M1 or M2 phenotypes (Zhou et al., 2020; Yan and Wan, 2021). The M1 phenotype, associated with classical activation, exerts pro-inflammatory effects, whereas the M2 phenotype primarily exerts protumor effects (Zhou et al., 2020). Specifically, M1 macrophages produce reactive oxygen species (ROS), reactive nitrogenous substances, and pro-inflammatory cytokines, such as TNF-α, IL-12, and IL-6. These substances stimulate Th1 immune reactions and improve the ability of CD8^+^ T cells to eliminate HPV-infected cells. In contrast, M2 macrophages inhibit CD8^+^ T-cell function by secreting IL-10 and TGF-β, promote regulatory T-cell (Treg) expansion, and create an immunosuppressive environment (Lin et al., 2019; Huang et al., 2021). Notably, macrophage polarization is a dynamic and complex process. Single-cell sequencing technology has suggested that the phenotypic landscape of macrophages within the microenvironment exhibits significant heterogeneity, extending beyond the simplistic binary classification of M1/M2. The distinction between M1 and M2 macrophages oversimplifies the intricate polarization process, which involves dynamic interactions between multiple cytokines, chemokines, and neighboring cells (Boutilier and Elsawa, 2021). Nevertheless, there is a paucity of research investigating the subtypes of macrophages under physiological or pathological conditions. This review focuses primarily on studies related to the M1 and M2 macrophage types. Natural killer (NK) cells defend against HPV infection. When activated, they produce perforin and granzymes, which induce apoptosis in infected cells, or secrete substantial amounts of inflammatory cytokines, such as interferon-γ and TNF-α. These cytokines inhibit viral replication and recruit other immune cells, including T cells and DCs, thus contributing to the development of HPV-specific adaptive immunity and improving viral elimination (Gutiérrez-Hoya and Soto-Cruz, 2021).

HPV uses multiple mechanisms to evade immune response and allow it to establish a persistent infection. Although the details of immune evasion are unclear, HPV proteins and certain cytokines are possibly involved (Westrich et al., 2017). The HPV16 E6 and E7 proteins inhibit immune cell function in the epithelium by decreasing macrophage-associated cytokines, such as TNF-α and macrophage inflammatory protein(MIP-3α), which blocks macrophage activation (Stern et al., 2000; Hacke et al., 2010; Bashaw et al., 2017). HPV infection impairs the antigen-presenting capacity of DCs by inhibiting monocyte differentiation into mature DCs (Lo Cigno et al., 2020). Another key mechanism involves the HPV E5 protein, which weakens NK cell responses by lowering CD1d expression in HPV16-infected cells, thus allowing them to evade immune detection and destruction. HR-HPV genotypes, such as HPV-16 and HPV-18, further suppress the host immune response by inhibiting type I IFN responses, which reduce immune cell activation (Doorbar et al., 2015; Lo Cigno et al., 2020). The E6 and E7 proteins of HPV16 and HPV18 can interfere with interferon regulatory factor function, leading to decreased IFN production. Additionally, these proteins disrupt the janus kinase-signal transducer and activator of transcription signaling pathway, which is crucial for interferon-mediated immune responses. This mechanism enables high-risk HPV types to evade innate immune surveillance and clearance (Woodby et al., 2018).

#### 4.2.2 Adaptive immunity

Upon infection, APCs process viral antigens and upregulate the expression of major histocompatibility complex molecules. These processed antigens are internalized by DCs via phagocytosis, after which DCs migrate to lymphoid tissues to activate adaptive immunity by secreting inflammatory cytokines, such as IL-1α, IL-1β, IL-6, TNF-α, and IL-12 (Chi et al., 2024). T cells can be further classified into helper T cells (Th), Tregs, and cytotoxic T cells. Th cells are subdivided into Th1, Th2, and Th17 subsets (Bordignon et al., 2017). Th1 cells produce IL-2, a key cytokine involved in protective immune responses, whereas Th2 cells produce IL-10, which may contribute to disease progression (Johansson and Lycke, 2003). The ratio of IL-2 to IL-10 reflects the Th1/Th2 immune response balance. Typically, a Th1-dominant state supports effective immunity; however, a shift toward Th2 dominance may lead to immunosuppression (Zheng et al., 2019). In B cell-mediated humoral immunity, secretory IgA (SIgA) and IgG are the principal effector molecules. SIgA is particularly important for mucosal defense, helping block pathogen entry in the reproductive tract (Dinesh et al., 2020). Meanwhile, T cell-mediated immunity is crucial for combating HPV. CD4^+^ T cells function as helper T cells, whereas CD8^+^ T cells function as cytotoxic or suppressor T cells. Patients with HR-HPV infections and cervical lesions exhibit reduced CD4^+^/CD8^+^ T cell ratios. Notably, CD4^+^ T cell levels are significantly higher in patients with CIN I than those with CIN II or III (Walch-Rückheim et al., 2015). Furthermore, the cervical microenvironment shows progressive changes with disease advancement: IL-2 concentrations decrease, whereas IL-10 production rises. This increase in IL-10 correlates with HPV infection severity, likely because of HPV proteins E2, E6, and E7 enhancing IL-10 gene transcription. Such elevated IL-10 expression may promote viral persistence and epithelial cell transformation, establishing a vicious cycle that supports carcinogenesis (Berti et al., 2017; Min et al., 2018). Furthermore, IL-10 may enhance the proliferation and cytotoxic function of HPV-specific CD8^+^ T lymphocytes induced by IL-2, potentially facilitating HPV clearance and protecting against cervical neoplasia (Farzaneh et al., 2006). Additionally, studies have reported increased IL-6 concentrations in HPV-positive individuals, with levels rising alongside cervical lesion severity. The proposed mechanism involves HPV E6/E7 proteins activating the IL-6/STAT3 signaling pathway, which mediates STAT3 phosphorylation in infected cells. This, in turn, enhances HPV E6/E7 protein expression, thereby promoting cervical tumor progression (Hao et al., 2020; Bonin-Jacob et al., 2021). Additionally, specific bacterial species within the microbiota may influence local immune responses and thereby potentially affect the progression of HPV-related diseases (Sims et al., 2021). VMB characterized by *Lactobacillus* depletion, elevated pH, and dysbiosis show increased levels of pro-inflammatory cytokines, such as IL-1β, IL-15, and TNF-α, as well as regulatory cytokines IL-12 and growth factor FGF2. These markers may mediate immune responses and chronic inflammation (Łaniewski et al., 2024). *Lactobacillus* in the vagina is negatively correlated with the expression of IL5/IL13 and TNFα but positively correlated with the expression of IL2 and IL12, which may mediate CC onset and progression (Yang et al., 2024b). Elevated levels of TLR7 and TLR9 have been detected in the cervical cells of BV-positive women infected with HPV, leading to the production of IFN and inflammatory cytokines, thereby causing tissue damage (Fracella et al., 2022).

Immunoglobulins are synthesized by B lymphocytes after antigen-stimulated proliferation and differentiation into plasma cells, which subsequently bind to specific antigens. Among them, IgA controls humoral immunity, whereas large amounts of IgG have been detected in the vagina in cases of persistent HPV infection (Dinesh et al., 2020). SIgA is the key effector molecule of the mucosal immune system. SIgA-mediated agglutination offers improved trapping potency, compared with IgG (Chen et al., 2015). Furthermore, it is normally expressed at low levels in the vagina. However, when the vaginal flora is dysbiotic, changes in bacterial metabolites can reduce SIgA degradation. Contrarily, immune responses triggered by pathogenic bacteria can increase local SIgA synthesis (Dinesh et al., 2020). SIgA secretion increases during mild vaginal infections but decreases in severe infections (Agarwal et al., 2010). Zheng et al. hypothesized that SIgA prevents pathogens from adhering to the cell surface in early-stage lesions, binds to microorganisms on mucosal surfaces, neutralizes viruses, and inactivates them by altering their conformation or blocking binding sites. This results in anti-infective effects and a reduction in SIgA concentration in the early stages of disease. In advanced stages, characterized by persistent HPV infection alongside severe vaginal flora imbalance, H_2_O_2_-producing Lactobacilli disappear and IgA protease secretion decreases (Li et al., 2024). This prevents the dissociation of disulfide bonds in the SIgA hinge region, resulting in elevated SIgA levels (Zheng et al., 2019).

### 4.3 Impact of vaginal microecological dysregulation on gene integration and transcription

The relationship between vaginal microecology and HPV infection involves intricate biological processes, particularly viral gene integration and transcription. Shifts in vaginal microecology may affect HPV infection development, particularly by playing a key role in viral gene integration and transcriptional regulation (Tian et al., 2022a).

Upon HPV entry into the host cell, its gene integration and transcription processes begin silently. The HPV genome consists of early (E) and late (L) gene regions. During gene integration, HPV DNA fragments are randomly inserted into the host genome, and their location often determines subsequent cellular transformation. When key oncogenes, such as p53 and Rb, serve as integration sites, HPV-derived transcripts may impair their normal activities, disrupting cell cycle regulation and apoptosis (Doorbar et al., 2012; Templeton and Laimins, 2023). The transcription of the E6 and E7 genes produces the corresponding E6 and E7 proteins, which bind to p53 and Rb, respectively. This binding leads to protein degradation, rendering cells more susceptible to uncontrolled proliferation (Xing et al., 2024). After HPV gene integration, viral gene transcription is regulated by host cell transcription factors. An imbalanced vaginal microecology may cause chronic inflammation, stimulating cytokine production. These cytokines activate signaling pathways that indirectly influence HPV promoter regions. This activation upregulates the transcription of key genes, such as NF-κB and AP-1, increasing viral protein synthesis and the risk of cellular lesions (Cruz-Gregorio and Aranda-Rivera, 2021). Furthermore, vaginal microecological disruption induces high levels of oxidative stress, generating ROS that cause double-stranded breaks in both the host genome and viral DNA. This process facilitates viral integration into host cells for replication and transformation. Through this mechanism, the HPV E6 protein suppresses the expression of E1 and E2 proteins, leading to dysregulated E6 and E7 transcription, unchecked viral proliferation, and a significant reduction in apoptosis (Adnane et al., 2018; Szymonowicz and Chen, 2020; Łaniewski and Herbst-Kralovetz, 2021).

In summary, vaginal microecology and HPV infection are intricately linked at the levels of gene integration and transcription. A deeper understanding of these mechanisms may provide novel avenues for the prevention, diagnosis, and treatment of HPV-related diseases.

### 4.4 Impact of cervicovaginal microbial metabolites

Metabolic dysregulation is an emerging hallmark of cancer, and metabolomics is increasingly being explored to identify specific biomarkers. Metabolomic analysis enables the rapid and precise detection of metabolites, making it highly valuable for studying cervical lesions and CC pathogenesis (Yang et al., 2024a).

Lactic acid, a metabolite produced by *Lactobacillus* plays a crucial role in HPV infection. It enhances cervical mucus’s ability to capture viral particles and inhibits HPV entry into basal cells (Pawar and Aranha, 2022). However, the antibacterial and anticancer effects of lactic acid depend on its type (D-lactic acid vs. L-lactic acid). CST I and II are typically dominated by D-lactic acid-producing *L. crispatus* or *L. gasseri*, forming a stable acidic environment. In contrast, CST III is primarily characterized by L-lactic acid-producing *L. iners*, resulting in an unstable acidic environment prone to dysbiosis. Additionally, the lack of other antimicrobial molecules (such as H_2_0_2_) further diminishes the defensive function of the vaginal microenvironment. This state is strongly associated with persistent HPV infection and recurrent BV. During CST IV, the microbiota becomes dysregulated, with an increase in anaerobic bacteria and a significant rise in vaginal pH (>4.5). The decrease in D-lactic acid concentration further weakens antiviral capacity (Borgogna et al., 2020; Dong et al., 2024). H_2_O_2_ impedes the progression of cervical lesions by selectively inducing apoptosis in malignant cells and denaturing bacterial proteins (Krüger and Bauer, 2017; Denys et al., 2019). The vulvovaginal metabolic profiles of HPV-infected women differ significantly from those of healthy controls in terms of lipid metabolism and amino acid metabolism, based on calculated metabolomic scores (Ilhan et al., 2019). Lipid metabolism is strongly related to genital inflammation and cervical lesions, with notably higher lipid accumulation in patients with high-grade CIN and CC because of its role in promoting cell proliferation and membrane synthesis via oncogene activation (Alvarez-Sieiro et al., 2016). Patients with HSIL show significantly elevated levels of acetylated phospholipids, sphingomyelins, phosphatidylcholine, and long-chain polyunsaturated fats. Similarly, 3-hydroxybutyrate, eicosapentaenoic acid esters, and oleic acid esters are markedly increased in patients with CC (Ilhan et al., 2019). Acetylated phospholipids and long-chain polyunsaturated fatty acids act as precursors to inflammatory mediators and may induce abnormal gene expression in cervical cells (Bokulich et al., 2022). Short-chain fatty acids (SCFAs), which are key microbial metabolites in the vaginal environment, regulate local immune responses by modulating vaginal epithelial cell function. SCFA concentrations are elevated in the vaginal tract of patients with BV. High SCFA levels may induce vaginal epithelial cells to secrete pro-inflammatory cytokines, impairing normal antiviral immune function. Additionally, they may disrupt the integrity of the vaginal barrier by affecting tight junction proteins in vaginal epithelial cells, thereby increasing susceptibility to HPV (Mirzaei et al., 2023). Changes in VMB and pH are influenced by amino acid metabolism. HPV infection is associated with reduced levels of key metabolites, including nicotinamide, succinate, and dipeptides (e.g., cysteinylglycine and cysteinyl) (McKenzie et al., 2021) as well as both oxidized and reduced glutathione (Borgogna et al., 2020). The total depletion of glutathione may contribute to oxidative stress, leading to irreversible cervical cell damage and promoting HPV persistence and carcinogenesis (Lebeau et al., 2022). Furthermore, ammonia produced by anaerobic bacterial metabolism and carcinogenic amyl nitrite have been detected in the vaginal environment of patients with BV. These compounds can stimulate the release of inflammatory cytokines, such as IL-1β and IL-8, which may interact with HPV and other factors to induce pathological changes in cervical epithelial cells. This process weakens immune defenses against HPV infection, whereas carcinogenic nitrosamines increase the likelihood of DNA damage (Wang et al., 2020b). Lactic acid produced by *Lactobacillus lactis* not only regulates vaginal pH but also indirectly affects nucleotide metabolism. The acidic environment can inhibit certain phosphatases involved in nucleotide phosphorylation and modification, affecting deoxynucleotide triphosphate (dNTP) production. This limitation in dNTP availability may restrict HPV replication (Ilhan et al., 2019; Fan et al., 2024).

Future research should further explore the association between vaginal microbial metabolites, HPV infection, and cervical carcinogenesis. Investigating targeted interventions in specific metabolic pathways may highlight novel approaches for disease prevention and management.

## 5 VMB-based diagnosis and treatment of HPV-related cervical diseases

Currently, effective solutions for HPV infection and low-grade cervical lesions are lacking. Surgical resection, radiotherapy, and chemotherapy are commonly utilized for high-grade lesions; however, these methods have drawbacks, such as fertility impairment and severe adverse effects (Kusakabe et al., 2023). Considering the close association between VMB and HPV infection as well as cervical lesions, VMB modulation has become a growing focus of research in recent years. In terms of early diagnosis, dynamic changes in vaginal microbial diversity may serve as potential biomarkers. High-throughput sequencing-based dynamic monitoring of vaginal microbiota, combined with HPV genotyping or metabolomic analysis (e.g., detection of lactic acid and SCFA levels), can assess the degree of microbial imbalance and aid in identifying high-risk populations (Kudela et al., 2021).

The core of prevention and treatment strategies lies in maintaining or restoring vaginal microecological homeostasis. The efficacy of L1 protein virus-like particle-based vaccines has been well-documented (Mlynarczyk-Bonikowska and Rudnicka, 2024); nonetheless, HPV vaccines do not protect against all HPV types that may develop into CC. Therefore, even vaccinated individuals must undergo regular cervical screenings (Williamson, 2023). Two key therapeutic strategies modulate the vaginal microbiota: probiotics and vaginal microbiome transplantation (VMT) (Zhang et al., 2024). The topical application of probiotics or prebiotics can enhance the vaginal acidic environment, inhibit pathogen colonization, and strengthen mucosal immune barrier function, thereby reducing HPV infection risk. *Lactobacillus* is the most commonly used probiotic for microbial modulation, followed by Bifidobacterium (Huang et al., 2024). Both oral or vaginal administration of probiotics, including *L. paracasei* and *L. rhamnosus*, can significantly increase HPV clearance rates (Huang et al., 2022). Chen et al. demonstrated that a multi-strain *Lactobacillus* probiotic combination significantly reduced pro-inflammatory cytokine levels (IL-1β and TNF-α) and immune infiltration (neutrophils, lymphocytes, and monocytes) in rat uteri. Hence, the anti-inflammatory properties of probiotics may partially explain their ability to aid HPV clearance (Chen et al., 2021). Bifidobacteria may further enhance anti-tumor immunity and the efficacy of immunotherapy (Kudela et al., 2021). *In vitro* experiments showed that co-culturing HPV-16-infected SiHa cells with Bifidobacteria reduced HPV E6/E7 mRNA levels (Curty et al., 2019). VMT involves transplanting healthy microbiota from a donor’s vagina into a patient’s vagina and holds promise for VMB improvement (Ma et al., 2019). However, current research on VMT remains limited. Some studies suggest that VMT requires specific vaginal environmental conditions in recipients as well as stringent donor microbiota health criteria, such as the absence of drug-resistant microbes or hidden pathogens in the donor’s microbiome (Gargiulo Isacco et al., 2023). Thus, further research is needed to determine its efficacy and potential adverse effects.

Additionally, multiple novel HPV therapies are currently under investigation. These include inhibitors targeting E1, E5, E6, and E7 proteins, L1 protein-based drugs, plant-derived medications, and therapeutic vaccines. These approaches aim to provide more effective treatment options by either directly inhibiting viral proteins or enhancing the host immune response (Mlynarczyk-Bonikowska and Rudnicka, 2024).

## 6 Summary

The vaginal microecosystem is a dynamic and balanced system, and alterations in this environment are closely associated with HPV infection. An imbalance in the VMB not only increases HPV infection risk but also impedes viral clearance, creating a vicious cycle. Restoring microbiome balance may improve HPV clearance rates and reduce the incidence of cervical lesions and cancer. Advancements in high-throughput sequencing and bioinformatics are progressively uncovering the mechanisms underlying the association between VMB and HPV clearance. Additionally, the development and clinical application of microbiota-based therapeutics for vaginal infections may provide novel treatment strategies for gynecological conditions, such as HPV infection. In conclusion, studying the VMB enhances the understanding of infections in the female reproductive tract and presents novel opportunities for CC prevention and management. Future large-scale prospective studies are essential to elucidate the composition and role of the vaginal microbiome in cervical lesion progression. As research continues to evolve in this field, further breakthroughs are expected.
